# Decanoic acid reduces extracellular prostaglandin accumulation, inhibits MRP4 transport, and enhances low-dose dexamethasone effects in IL-1β-stimulated human osteoarthritis synoviocytes

**DOI:** 10.3389/fmed.2026.1876378

**Published:** 2026-10-20

**Authors:** Jason Williams, Melissa A. Hausburg, Gregory W. Thomas, Raphael Bar-Or, Kaysie Banton, David Bar-Or

**Affiliations:** 1 Trauma Research, Swedish Medical Center, Englewood, CO, United States; 2 Trauma Research, Lutheran Medical Center, Wheat Ridge, CO, United States; 3 Trauma Research, Wesley Medical Center, Wichita, KS, United States; 4 Trauma Research, South Texas Health System, McAllen, TX, United States

**Keywords:** ABCC4, decanoic acid, dexamethasone, MRP4, osteoarthritis, prostaglandin E2, specialized pro-resolving mediators, synoviocytes

## Abstract

**Objective:**

Persistent extracellular prostaglandin signaling contributes to synovial inflammation and pain in osteoarthritis (OA), although prostaglandin E2 (PGE2) can also have context-dependent homeostatic functions. Decanoic acid (DA) is a 10-carbon medium-chain fatty acid with reported peroxisome proliferator-activated receptor-modulating and anti-inflammatory activity. Its amphipathic carboxylate structure also made it an exploratory candidate for interaction with the hydrophobic organic-anion binding region of multidrug resistance protein 4 (MRP4/ABCC4). We tested whether DA changes prostaglandin compartmentalization and enhances the effect of very-low-dose dexamethasone (Dex) in interleukin-1β (IL-1β)-stimulated human OA fibroblast-like synoviocytes.

**Methods:**

Primary human osteoarthritis fibroblast-like synoviocytes were stimulated with IL-1β and treated with DA, Dex, or their combination. Extracellular and intracellular prostaglandins were quantified by competitive ELISA. MRP4 inhibition was evaluated in inside-out HEK293 membrane vesicles overexpressing human MRP4 using ATP-dependent DHEAS transport, with MK-571 as the positive inhibitor control. Molecular docking and UHPLC-MS lipidomics were also performed. Replicates were independently cultured wells or pellets from the same donor

**Results:**

DA reduced extracellular PGE2 and increased intracellular PGE2. With a fixed 0.1 nM Dex background, the fitted DA IC50 changed from 19.42 μM (95% CI, 11.14–32.49 μM) to 8.62 μM (95% CI, 4.69–15.26 μM) with an *F*-test *P* = 0.0402, indicating Das ability to potentiate the inhibitory effect of sub-nanomolar DEX. DA also reduced extracellular PGD2 and PGI2. In vesicles, DA inhibited MRP4-mediated DHEAS transport in a concentration-dependent manner (calculated IC50, approximately 33.86 μM), but PGE2 transport was not measured directly. Docking placed DA near the MK-571 reference pose. DA was also associated with increased 17(S)-HDHA and maresin 2 (MaR2).

**Conclusions:**

In this single-donor proof-of-concept model, DA altered prostaglandin distribution, inhibited MRP4 probe-substrate transport, and enhanced extracellular prostaglandin suppression in the presence of low-dose Dex. The findings are consistent with, but do not prove, an MRP4-associated contribution to prostaglandin export. The increases in 17(S)-HDHA and MaR2 are associative and do not establish that intracellular PGE2 retention caused resolution signaling. Multi-donor validation, direct PGE2 transport studies, genetic MRP4 experiments, and intra-articular pharmacokinetic and safety studies are required.

## 1 Introduction

Osteoarthritis (OA) is a chronic, degenerative joint pathology defined by cartilage erosion, synovial inflammation, pain, stiffness, and a progressive loss of function. While mechanical trauma and repetitive loading contribute to its onset, the clinical syndrome is increasingly recognized as a biologically active process involving dysregulated inflammatory signaling, altered lipid mediator metabolism, and the activation of chondrocytes and synoviocytes, alongside impaired inflammatory resolution (1, 2). The burden on patients and healthcare systems is substantial and will likely intensify as populations age and the demand for arthroplasty increases (3, 4). Current management strategies remain primarily symptomatic, utilizing nonsteroidal anti-inflammatory agents, intra-articular corticosteroids, and hyaluronic acid preparations, with joint replacement as the final intervention; notably, despite ongoing investigation into ion-channel, regenerative, biologic, gene-based, and cell-based approaches, effective disease-modifying therapies remain limited (5–7).

A major pharmacologic target in OA inflammation is the prostanoid pathway. Cyclooxygenase-1 and cyclooxygenase-2 convert arachidonic acid into prostaglandin H2, which is subsequently metabolized into prostaglandins and thromboxanes, including PGE2, PGD2, PGI2, and TXB2 (8–11). In OA-relevant cells, IL-1β, TNF-α, and mechanical stress induce COX-2 and prostaglandin E synthase, thereby increasing PGE2 production (12–14). PGE2 acts through EP1-EP4 receptors to regulate inflammatory signaling, vascular permeability, matrix degradation, apoptosis, nociceptor sensitization, and cytokine production (13, 15–17). However, the effect of PGE2 is context dependent: its biological consequences vary by cell type, receptor, concentration, and disease stage, and emerging work also supports homeostatic or chondroprotective functions in cartilage-related systems (18, 19).

Prostaglandins also participate in the transition from inflammation to resolution. During acute inflammation, prostaglandins participate in lipid mediator class switching, promoting the transition from pro-inflammatory to resolution and enabling production of specialized pro-resolving mediators (SPMs), including resolvins, protectins, lipoxins, and maresins (20, 21). SPMs reduce leukocyte recruitment, improve efferocytosis, support tissue repair, and may counter synovitis and cartilage injury in arthritis models (22–25). This dual role creates a therapeutic challenge: suppressing prostaglandin synthesis can reduce inflammatory pain and swelling, but complete upstream blockade may also impair normal resolution stage.

Before extracellular PGE2 can activate cell-surface EP receptors, it must leave the producing cell. Multidrug resistance protein 4 (MRP4), also known as ATP-binding cassette subfamily C member 4 (ABCC4), is an ATP-dependent transporter capable of exporting prostanoids, cyclic nucleotides, drugs, and other organic anions (26). Recent structural studies have clarified the molecular basis of MRP4 substrate transport and prostaglandin efflux, including a hydrophobic substrate pocket relevant to PGE2 and known inhibitors (27, 28). MRP4 may therefore regulate prostaglandin distribution between intracellular and extracellular compartments. In principle, reducing export could attenuate extracellular receptor activation while preserving intracellular prostaglandin pools; however, whether retained prostaglandins remain biologically beneficial or are redirected into resolution pathways requires direct experimental testing.

Glucocorticoids such as Dex and triamcinolone acetonide remain widely used to reduce OA symptoms because of their robust anti-inflammatory effects (29, 30). Yet repeated intra-articular corticosteroid exposure has raised concerns regarding cartilage matrix damage, chondrocyte senescence, and adverse effects on chondrocyte morphology, particularly at higher or repeated doses (31–33). A steroid-sparing strategy that maintains anti-inflammatory potency while reducing steroid exposure would therefore have translational value.

DA was selected as an exploratory compound for several reasons. It is a naturally occurring 10-carbon medium-chain saturated fatty acid and a direct, partial modulating ligand of peroxisome proliferator-activated receptor-*γ*, with weaker reported interactions with other PPAR isoforms (34). Capric acid/DA has also shown anti-inflammatory effects in bone-relevant cell systems, including suppression of receptor activator of NF-*κ*B ligand-induced NF-*κ*B signaling in osteoclast precursors (35). In addition, its compact hydrophobic chain and ionizable carboxylate provide an amphipathic structure that could plausibly access an organic-anion-binding pocket. DA was not previously established as an MRP4 inhibitor; its evaluation here was therefore hypothesis-generating rather than confirmatory.

We tested whether DA reduces extracellular prostaglandin accumulation in IL-1β-stimulated human OA fibroblast-like synoviocytes and whether a fixed, minimally active concentration of Dex enhances that effect. We further examined whether reciprocal changes in extracellular and intracellular PGE2, inhibition of MRP4-mediated DHEAS probe transport, and molecular docking were consistent with an MRP4-associated transport hypothesis. Finally, we assessed whether DA treatment was associated with changes in inflammatory and resolution-associated oxylipins. Because the cell experiments used one donor and the transport assay did not use PGE2 as the probe substrate, the study was designed and interpreted as preliminary mechanistic evidence rather than definitive proof of prostaglandin transport inhibition.

## 2 Materials and methods

### 2.1 Cell culture

Human primary fibroblast-like synoviocytes (HFLS) isolated from OA cadaveric knee tissue from one donor (Articular Engineering LLC) were cultured at 37 °C in 5% CO2 in DMEM/F12 (Gibco, 10565-018) supplemented with 10% fetal bovine serum (Gibco, 12483020). All cell-based experiments reported in this manuscript, including the lipidomic experiment, used cells from this same donor preparation. Unless otherwise specified, n denotes independently cultured wells or cell pellets from this donor and does not denote independent human donors.

To induce inflammatory activation, medium was replaced with identical medium containing 1 ng/mL IL-1β (Gibco, PHC0815) for 24 h. Treatment conditions received dexamethasone sodium phosphate (Fresenius Kabi, NDC 63323-0506; referred to as Dex) at final concentrations of 0.5 nM or 0.1 nM and sodium decanoate (Tokyo Chemical Industry, D0024; referred to as DA) at final concentrations ranging from 0.39 to 200 μM, separately or in combination as specified for each experiment. After 24 h, medium was collected, aliquoted, and stored at −80 °C. Cells were washed with cold phosphate-buffered saline, pH 7.4 (Gibco, 10010-023), and lysed in 0.02% Triton X-100 containing 1 ×  Halt Protease Inhibitor Cocktail (Thermo Scientific, 78429). Lysates were sonicated for 5 min in a bath sonicator and stored at −80 °C until analysis.

### 2.2 Quantification of prostaglandins

Prostaglandins were quantified using competitive ELISAs according to the manufacturers’ protocols. PGE2 was quantified using the Prostaglandin E2 Express ELISA kit (Cayman Chemical, 514010). Cell culture media samples were diluted 30-fold in ELISA buffer and cell lysates were diluted 10-fold. PGD2 was quantified using the Prostaglandin D2-MOX ELISA kit (Cayman Chemical, 512011), with methoximated media samples diluted 2-fold. PGI2 was quantified using the Prostacyclin ELISA Kit (Novus Biologicals, NBP2-62171), with media samples diluted 25-fold.

### 2.3 MRP4 vesicular transport assay

MRP4 transport inhibition studies were conducted by SOLVO/Charles River Laboratories Hungary using inside-out membrane vesicles prepared from HEK293 cells stably overexpressing human MRP4. Vesicular transport assays were performed according to SOLVO vesicular transport inhibition protocols (36, 37). Human MRP4 vesicles were incubated with 0.5 μM dehydroepiandrosterone sulfate (DHEAS) probe substrate, 50 μg protein per well, and either MgATP or AMP background control. DA was tested at final concentrations of 7.81, 15.63, 31.25, 62.5, 125, 250, and 500 μM; MK-571 was used as the positive inhibitor control. Reaction mixtures were pre-incubated with DA or control inhibitor for 15 min at 37 °C, initiated by addition of MgATP, incubated for 1.5 min, quenched with ice-cold washing buffer, filtered through glass fiber filters, washed, dried, and quantified by liquid scintillation counting. ATP-dependent transporter-specific accumulation was calculated relative to vehicle control, with vehicle defined as 100% activity and absence of transporter activity defined as 0%. IC50 values were calculated by nonlinear regression using a four-parameter curve-fit model. Please see Supplemental Methods for a more detailed method.

### 2.4 Molecular docking

Molecular docking was performed with AutoDock Vina 1.2.5 to compare predicted binding of MK-571, PGE2, DA, cAMP, caprylic acid, and lauric acid to human MRP4 (38, 39). Ligands were prepared from SMILES representations using molscrub at pH 7.4. The receptor was prepared from the cryo-EM structure of nanodisc-reconstituted wild-type human MRP4 in the inward-facing conformation (PDB: 8BJF). Docking was restricted to the known hydrophobic pocket of MRP4 centered at 159, 156, 145 with box dimensions of 20 × 20 × 100 Å, and poses were visualized in ChimeraX.

### 2.5 Lipidomic analysis

For lipidomic analysis, HFLS cells were seeded in 6-well plates at 500,000 cells per well and stimulated for 6 h with 1 ng/mL IL-1β. Cells were treated with 0.1 nM Dex and/or 100 μM or 200 μM DA, alone or in combination. After 6 h, cells were washed with cold PBS, harvested gently with TrypLE (Gibco, 12605-028), pelleted, frozen at −80 °C, and submitted for lipidomic analysis. Four independently cultured cell pellets from the same donor were analyzed per condition.

Lipidomics was performed at the Metabolomics Core Facility of the University of Colorado Anschutz Medical Campus. Cell pellets were extracted with cold 5:3:2 methanol:acetonitrile:water, vortexed for 30 min at 4 °C, centrifuged, and analyzed by UHPLC-MS using a 1.7 μm, 2.1 × 100 mm Acquity BEH column and a 7-min gradient as previously described (40, 41). Deuterated oxylipin standards were run separately to determine retention times. Due to limitations of the standard set and co-elution, some oxylipins could not be differentiated when they shared parent ion mass and retention time; for example, 8-isoPGE2, PGE2, and PGD2 shared a retention time of 2.9 min. For the list of Standards used please see Supplemental Methods.

Raw files were converted to mzXML using RawConverter. Metabolites were assigned based on intact mass, 13C isotope pattern, retention time, deuterated standards, and KEGG database matching. Peaks were integrated using Maven. Quality control was assessed using a technical mixture of all samples run at the beginning, middle, and end of each sequence. Statistical analyses were performed using MetaboAnalyst 6.0 and GraphPad Prism. Peak intensity values were log10 transformed before univariate testing.

### 2.6 Statistical analysis and replicate definitions

For cell-based ELISA experiments, bars represent the mean ± standard deviation of cultured wells from independent experiments from the single donor, with replicate counts stated in the figure legends. For lipidomics, log10-transformed data were analyzed by ordinary one-way analysis of variance (ANOVA) with Benjamini-Hochberg false discovery rate (FDR) correction (42). The MRP4 vesicle assay used three assay determinations per concentration and reported propagated error as supplied by the contract laboratory.

DA concentration-response curves were fit with a four-parameter logistic model. The fitted IC50 estimates and profile-likelihood 95% confidence intervals were 19.42 μM (11.14–32.49 μM) for DA alone and 8.62 μM (4.69–15.26 μM) for DA with 0.1 nM Dex. An extra-sum-of-squares *F* test was performed in Graphpad Prism using non-linear regression tool {log(inhibitor) vs. normalized response -variable slope}. Following this, the two Log IC50s were compared for significance to their best-fit.

## 3 Results

### 3.1 Low-dose Dex potentiates DA-mediated suppression of extracellular PGE2

We first defined the Dex dose range in IL-1β-stimulated primary OA synovial fibroblasts to identify a concentration suitable for combination testing. High Dex concentrations produced near-maximal suppression of extracellular PGE2: 100 nM and 10 nM Dex each inhibited PGE2 release by approximately 98% compared with IL-1β control, and 1 nM Dex inhibited PGE2 by approximately 73%. In contrast, 0.1 nM Dex produced only minimal inhibition. This submaximal 0.1 nM concentration was therefore selected for the primary DA-Dex combination experiment because it preserved dynamic range for detecting potentiation (Figure 1A).

**Figure 1:**
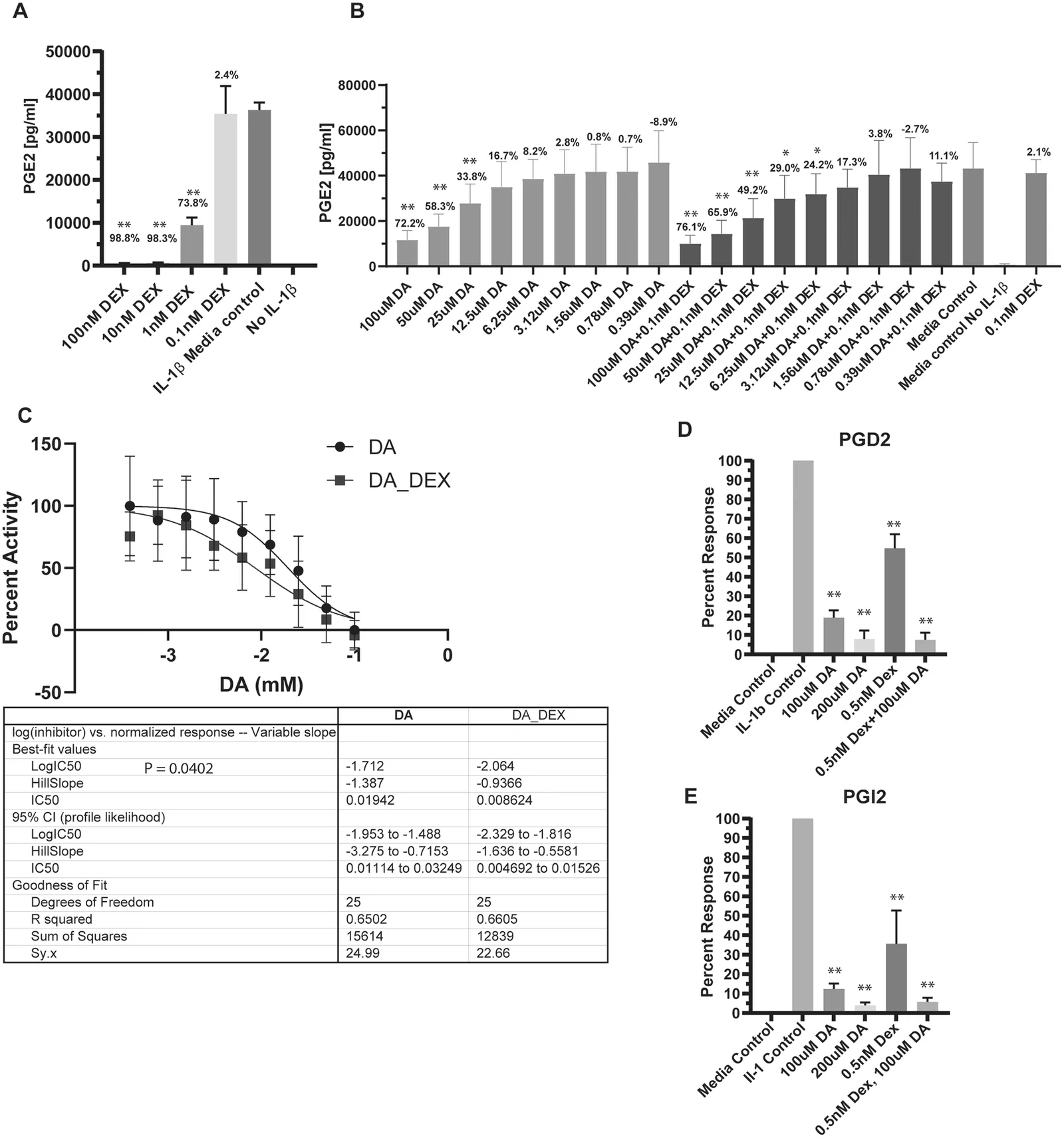
DA enhances low-dose Dex suppression of extracellular prostaglandins. **(A)** Dex concentration-response showing near-maximal extracellular PGE2 suppression at 10−100 nM Dex and minimal suppression at 0.1 nM Dex (*n* = 4). **(B)** DA concentration-response in the absence or presence of fixed 0.1 nM Dex. (*n* = 3). **(C)** Leftward shift in DA IC50 when combined with 0.1 nM Dex (*n* = 3). **(D,E)** Suppression of extracellular PGD2 and PGI2 by 100 μM DA, 0.5 nM Dex, and the DA-Dex combination (*n* = 4). Error bars represent Standard Deviation. *p *<* 0.01. **p *<* *0.*001 using One-way Anova with FRD method of Benjamini and Hochberg. *n* *=* within-donor independent experiment cultured wells per condition.

DA was then tested across a nine-point concentration range, alone or in the presence of fixed 0.1 nM Dex (Figure 1B). Across several mid-range DA concentrations, the combination produced greater inhibition of extracellular PGE2 than DA alone. At 25 μM DA, inhibition increased from 33.8% with DA alone to 49.2% with DA plus 0.1 nM Dex. At 12.5, 6.25, and 3.12 μM DA, addition of 0.1 nM Dex increased inhibition by 12.3, 16.0, and 14.4 percentage points, respectively. The combination of 50 μM DA plus 0.1 nM Dex achieved 65.9% inhibition, approaching the effect of 100 μM DA alone.

Four-parameter curve fitting yielded an IC50 point estimate of 19.42 μM for DA alone and 8.62 μM for DA plus 0.1 nM Dex (Figure 1C). The corresponding 95% confidence intervals were 11.14–32.49 μM and 4.69–15.26 μM, respectively. Comparing the two fitted logIC50 values using an extra-sum-of-squares *F*-test yielded *P* = 0.0402, indicating that the fitted concentration-response curves differed significantly and supporting DA-mediated potentiation of very-low-dose Dex.

### 3.2 DA and Dex reduce extracellular PGD2 and PGI2

To determine whether the DA-Dex effect extended beyond PGE2, we quantified extracellular PGD2 and PGI2. This second prostaglandin experiment used 0.5 nM Dex, not 0.1 nM Dex, and therefore should be interpreted separately from the PGE2 IC50/potentiation experiment above. PGH2 was not detectable in this synoviocyte model. Downstream prostaglandins PGD2 and PGI2 showed a pattern similar to PGE2. Dex at 0.5 nM inhibited PGD2 and PGI2 by 45% and 64%, respectively. DA at 100 μM inhibited PGD2 and PGI2 by 81% and 88%, respectively. Combination treatment with 100 μM DA plus 0.5 nM Dex further increased inhibition to 92% for PGD2% and 94% for PGI2 (Figures 1D,E). These data indicate that DA-Dex treatment reduces multiple extracellular prostaglandins, but the PGD2/PGI2 results should not be merged analytically with the 0.1 nM Dex PGE2 dose-response experiment.

### 3.3 DA increases intracellular PGE2 while reducing extracellular PGE2

Dex acts upstream in the eicosanoid pathway by suppressing phospholipase A2 and cyclooxygenase/prostaglandin E isomerase activity (43). To distinguish a simple reduction in synthesis from altered prostaglandin distribution, we compared PGE2 in culture medium and cell lysates. DA was tested at 100 and 200 μM, and the combination condition used 100 μM DA plus 0.5 nM Dex. DA reduced extracellular PGE2 by 80.6% and 96.6% at 100 and 200 μM, respectively. The combination reduced extracellular PGE2 by 97.16% (Figure 2A).

**Figure 2:**
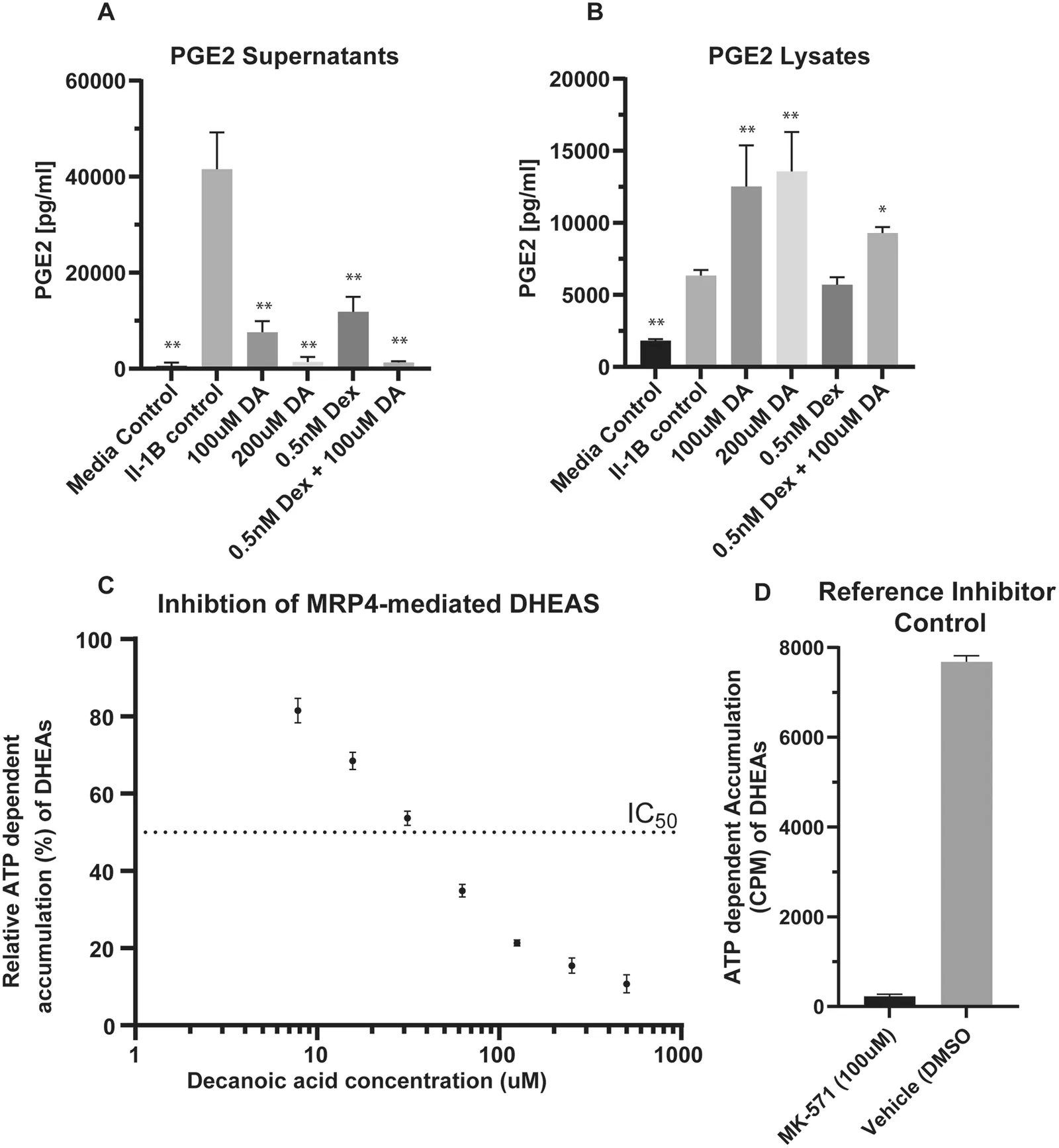
DA reduces extracellular PGE2, increases intracellular PGE2, and inhibits human MRP4-mediated transport. **(A)** Extracellular PGE2 after IL-1β stimulation and treatment with DA, Dex, or DA-Dex. **(B)** Intracellular PGE2 in the same experimental context, showing intracellular accumulation after DA treatment. **(C)** Concentration-dependent inhibition of ATP-dependent MRP4 vesicular transport by DA. **(D)** MK-571 positive-control inhibition of MRP4 transport. Error bars represent Standard Deviation. *p *<* 0.01. **p *<* *0.*001 using One-way Anova with FRD method of Benjamini and Hochberg. *n* *=* within-donor independent experiment cultured wells per condition.

In contrast, intracellular PGE2 increased by 49.4% with 100 μM DA and 53.3% with 200 μM DA relative to IL-1β control. In the DA plus Dex condition, intracellular PGE2 increased by 31.8% (Figure 2B). This reciprocal pattern is not explained by complete suppression of PGE2 synthesis alone and is compatible with altered export or intracellular handling. However, it is not transporter specific and does not by itself establish MRP4-dependent PGE2 retention.

### 3.4 DA directly inhibits human MRP4-mediated transport

MRP4/ABCC4 is a confirmed prostanoid efflux transporter and is structurally linked to prostaglandin export (26–28). We therefore tested DA directly in a human MRP4 vesicular transport inhibition assay. In this cell-free system, inside-out membrane vesicles from HEK293 cells overexpressing human MRP4 were incubated with DHEAS probe substrate in the presence of ATP or AMP background control. DA inhibited ATP-dependent MRP4 transport in a concentration-dependent manner (Figure 2C and Table 1). Relative ATP-dependent accumulation fell from 100% in vehicle control to 81.51% at 7.81 μM DA, 68.47% at 15.63 μM, 53.64% at 31.25 μM, 34.85% at 62.5 μM, 21.33% at 125 μM, 15.45% at 250 μM, and 10.72% at 500 μM. These values correspond to 18%, 32%, 46%, 65%, 79%, 85%, and 89% inhibition, respectively. The calculated IC50 was approximately 33.86 μM. MK-571, the positive-control MRP4 inhibitor, reduced relative accumulation to 2.98%, corresponding to 97% inhibition (Figure 2D). These results demonstrate that DA can inhibit MRP4-mediated transport of the DHEAS probe substrate at concentrations overlapping the active range in the synoviocyte experiments. Because DHEAS and PGE2 can interact differently with MRP4, the assay supports an MRP4-associated mechanism but does not directly demonstrate inhibition of prostaglandin transport.

**Table 1:** DA inhibits ATP-dependent human MRP4 vesicular transport of DHEAS probe substrate. Values are mean ± error propagation for *n* = 3. Relative inhibition was calculated against vehicle control. DHEAS was used as the probe substrate and MK-571 as the positive inhibition control.

Compound	Nominal concentration (μM)	ATP dependent accumulation (CPM)	Relative ATP dependent accumulation (%)	Relative inhibition (%)
		Average ± EP	Average ± EP	Average
	500.00	824.00 ± 176.65	10.72 ± 2.31	89
	250.00	1187.00 ± 148.03	15.45 ± 1.95	85
	125.00	1639.00 ± 52.35	21.33 ± 0.78	79
Decanoic Acid	62.50	2677.33 ± 114.64	34.85 ± 1.61	65
	31.25	4121.67 ± 118.92	53.64 ± 1.82	46
	15.63	5261.00 ± 139.79	68.47 ± 2.19	32
	7.81	6262.33 ± 215.91	81.51 ± 3.16	18
Vehicle (DMSO)	0.00	7683.33 ± 135.94	100.00 ± 2.50	0
MK-571	100.00	229.00 ± 42.25	2.98 ± 0.55	97

Average and error propagation (EP) calculated for *n* = 3.

### 3.5 Molecular docking places DA near the MRP4 inhibitor/substrate pocket

To evaluate whether DA could access a substrate- or inhibitor-relevant region of MRP4, docking was performed within the known hydrophobic pocket of MRP4 (27, 28). MK-571 had the most favorable mean predicted binding affinity (−9.222 kcal/mol) and was used as the positional reference pose (Figure 3A). PGE2 docked near MK-571, with a mean predicted affinity of −7.201 kcal/mol and a distance of 3.92 Å from the MK-571 pose (Figure 3B). DA had a weaker predicted affinity (−5.470 kcal/mol) but localized very close to the MK-571 reference pose (1.37 Å), the closest non-reference ligand among those tested (Figure 3C). cAMP showed a favorable predicted affinity (−8.362 kcal/mol) but docked farther from the MK-571 pose (9.63 Å) (Figure 3D). These data are consistent with the possibility that DA can occupy a relevant region of the MRP4 hydrophobic pocket, although docking should be interpreted as supportive rather than definitive evidence of binding.

**Figure 3:**
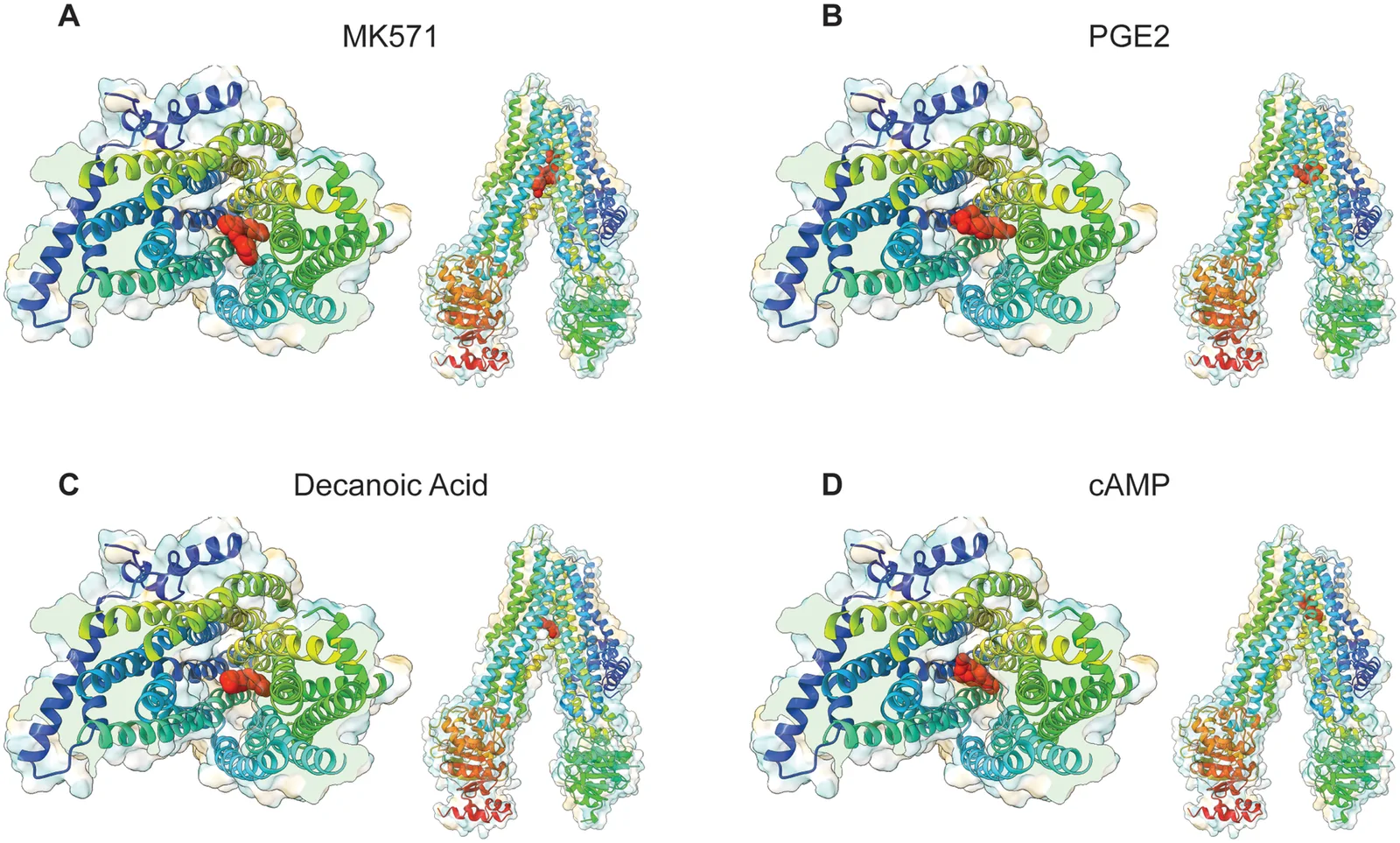
Molecular docking of DA and comparator ligands in the MRP4 hydrophobic pocket. Side and top-down views of **(A)** MK-571 reference pose. **(B)** PGE2 docking near the MK-571 reference pose. **(C)** DA docking close to the MK-571 reference pose. **(D)** cAMP docking farther from the MK-571 reference pose despite stronger predicted affinity than DA.

### 3.6 IL-1β induces a pro-inflammatory lipid mediator response

Evidence indicates that elevated levels of prostanoids, such as PGE2 and PGD2, help mediate the transition from pro-inflammatory to pro-resolving signaling. This process involves a specific category of oxylipins known as specialized pro-resolving lipid mediators (SPMs), which include resolvins, protectins, maresins, and lipoxins (21, 44) (Supplementary Figure S1). We next examined oxylipin profiles at 6 h after IL-1β stimulation. IL-1β induced a pronounced inflammatory lipid mediator response. UHPLC-MS profiling identified 34 high-confidence lipid mediators (Supplementary Figure S2), of which nine were significantly altered after IL-1β exposure (FDR < 0.05; Figure 4A). Notable increases included 15(S)-HETE (12.33-fold, FDR = 0.0008), 8-isoPGE2/PGE2/PGD2 (22.65-fold, FDR = 0.002), 11(S)-HETE/12(S)-HETE (3.93-fold, FDR = 0.002), 6-keto PGF1a (9.54-fold, FDR = 0.008), Lipoxin A4/13-14-dihydro-15-keto PGD2 (2.81-fold, FDR = 0.014), 5-iPF2a-VI/PGF2a/PGD1/PGE1 (14.37-fold, FDR = 0.040), and 9(S)-HODE (1.62-fold, FDR = 0.048). IL-1β also modestly increased selected resolution-associated mediators, including 17(S)-HDHA (3.89-fold, FDR = 0.007) and MaR2 (4.35-fold, FDR = 0.048), but these increases were smaller than the broader pro-inflammatory shift.

**Figure 4:**
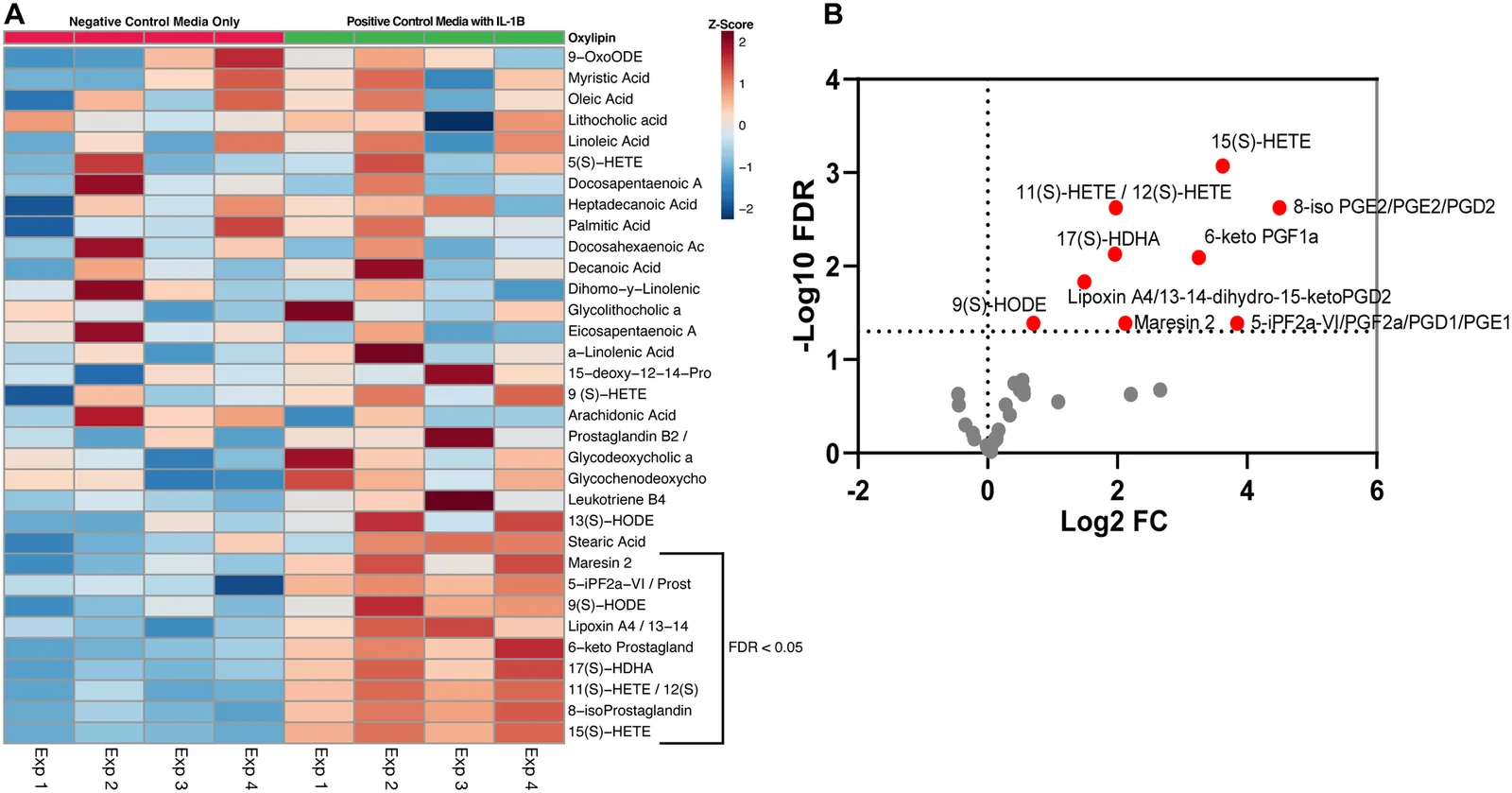
IL-1β-associated oxylipin changes in synoviocytes. **(A)** Heatmap with Z-scored relative abundances for each oxylipin. **(B)** Volcano plot depicting Log2(fold change) and -Log10(False discovery rate) of all oxylipins. Fold change was calculated between cells with and without IL-1β stimulation. Four independently cultured cell pellets from the same donor were analyzed per condition. Significant (FDR < 0.05) oxylipins are indicated by red circles.

### 3.7 DA-containing treatments increase 17(S)-HDHA and MaR2 pro-resolving lipid mediators

DA treatment increased production of resolution-associated lipid mediators in IL-1β-stimulated synoviocytes. At 100 μM and 200 μM DA, 17(S)-HDHA increased 5.14-fold (*p* = 0.0003) and 7.70-fold (*p* = 0.01), respectively (Figure 5B). MaR2 increased 5.8-fold (*p* = 0.003) and 8.70-fold (*p* = 0.02), respectively (Figure 5C). Dex alone did not increase 17(S)-HDHA or MaR2 compared with control conditions. Combination treatment with Dex and DA preserved the DA-associated increase in 17(S)-HDHA (5.07-fold, *p* = 0.0076) and MaR2 (4.98-fold, *p* = 0.0214). These findings show that DA-containing conditions were associated with increased concentrations of selected resolution-associated lipid mediators, whereas Dex alone did not reproduce this pattern. Additionally, the data do not establish that intracellular PGE2 retention caused the changes or that a complete lipid mediator class switch occurred.

**Figure 5:**
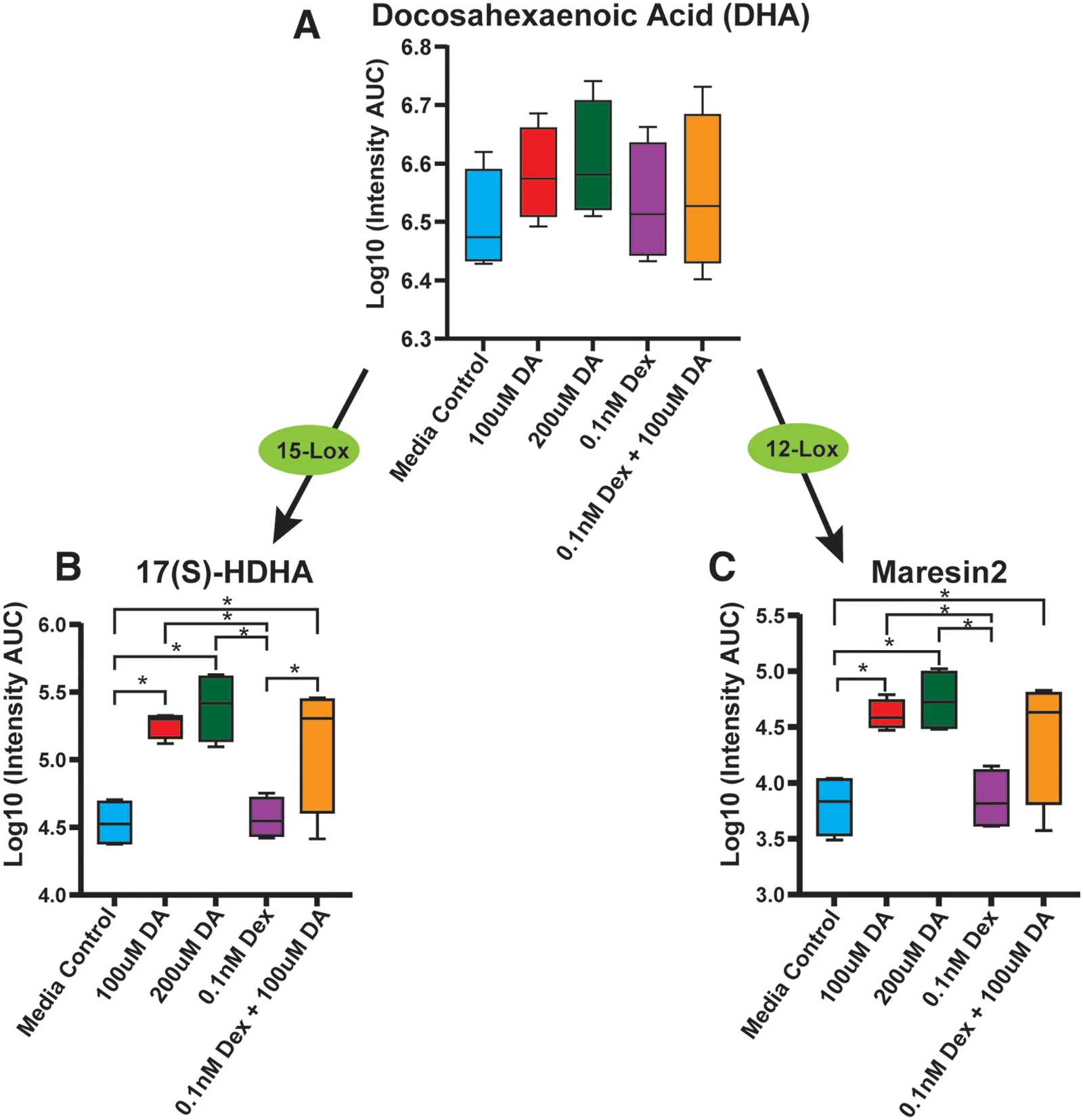
DA-associated increases in resolution-related oxylipins. Box and whiskers plot of Log10 transformed Intensity (AU) values **(A)** DHA. **(B)** 17(S)-HDHA. **(C)** Maresin2. (*n* = 4) Whiskers denote 10–90 percentile. *P < 0.05 using One-way Anova with FRD method of Benjamini and Hochberg. Four independently cultured cell pellets from the same donor were analyzed per condition.

## 4 Discussion

This study identifies MRP4-mediated prostaglandin export as a mechanistically important and therapeutically targetable control point in IL-1β-stimulated human OA synoviocytes. The central finding is that DA does not behave simply as a broad inhibitor of inflammatory lipid synthesis. Instead, DA reduces extracellular prostaglandin accumulation while increasing intracellular PGE2 (Figures 2A,B), potentially inhibits human MRP4-dependent transport in vesicles (Figures 2C,D and Table 1), and docks near the MRP4 hydrophobic inhibitor/substrate pocket (Figure 3). In parallel, DA increases selected resolution-associated lipid mediators, particularly 17(S)-HDHA and MaR2 (Figure 5).

The combination data are important because they suggest more than parallel anti-inflammatory activity. In the PGE2 concentration-response experiment, 0.1 nM Dex was deliberately chosen because it had minimal independent activity, avoiding the ceiling effect seen at 10–100 nM Dex (Figure 1A). Against that low Dex background, the DA IC50 decreased from 19.42 μM to 8.62 μM, and several adjacent mid-range DA concentrations showed higher inhibition in the combination condition than with DA alone (Figures 1B,C). This DA-Dex interaction is best described from the current summarized dataset as potentiation, with synergy-consistent features that require replicate-level confirmation.

The current experiment was designed to determine whether a minimally active fixed Dex concentration shifted the DA concentration response curve rather than to generate a complete DA-by-Dex interaction surface. Although a *post hoc* combination index could be estimated by interpolation from the fitted single-agent curves, only one Dex concentration was represented throughout the combination series. Any such estimate would therefore be highly model-dependent and would not characterize the interaction across multiple dose ratios or effect levels. We consequently interpret the leftward shift in the DA concentration response curve as pharmacologic potentiation and do not assign a formal combination index or claim definitive synergy from the present synoviocyte dataset.

In the live-cell experiments, DA decreased extracellular PGE2 but increased intracellular PGE2, a pattern difficult to explain by inhibition of PLA2, COX-2, or terminal PGE synthase alone (Figures 2A,B). If DA primarily blocked prostaglandin synthesis, intracellular PGE2 would be expected to fall together with extracellular PGE2. Instead, the reciprocal relationship suggests that PGE2 continues to be generated but is retained intracellularly. The vesicular transport assay then provided direct biochemical support for this interpretation: DA inhibited ATP-dependent MRP4-mediated DHEAS probe transport in a concentration-dependent manner, with strong inhibition in the 125–500 μM range and measurable inhibition even near 31.25 μM (Figure 2C and Table 1). The MK-571 positive control confirmed that the assay was detecting MRP4-dependent transport (Figure 2D). These results move the study past simple correlation and point to MRP4 inhibition as the potential mechanism.

Evidence that the extracellular PGE2 response is not restricted to synoviocytes has now been reported by our group in primary human chondrocytes. In a separate study using two donor-specific chondrocyte models, we show that DA and Dex each reduced IL-1β-induced extracellular PGE2, whereas co-treatment increased potency and maximal inhibition. Longer-term experiments in an OA chondrocyte model also suggested complementary effects on MMP13 expression and PPAR*γ* activity (45). These findings support the presence of a DA-Dex interaction in another OA-relevant joint cell type. They do not, however, establish that the MRP4-associated transport mechanism or resolution-associated lipidomic effects observed in the present synoviocyte study are identical in chondrocytes. Future studies should extend MRP4-specific transport and lipidomic analyses to chondrocytes and should use additional primary synoviocyte donors and complementary non-primary synoviocyte models.

DA may also act through mechanisms independent of MRP4. It is a direct modulating ligand of PPAR*γ* (34), and has been reported to suppress NF-*κ*B signaling in osteoclast-lineage cells (35). Such effects could alter prostaglandin-synthetic enzymes, fatty-acid handling, lipoxygenase pathways, membrane organization, or inflammatory transcription. The docking result (Figure 3) is supportive but cannot distinguish competitive transport inhibition from membrane-mediated or transcriptional effects. Comparisons with structurally related medium-chain fatty acids, selective MRP4 inhibitors, and genetic MRP4 loss-of-function would help determine the relative contribution of these mechanisms.

The 100–200*μ*M DA concentrations used in this study correspond to approximately 17–34 μg/mL DA. In healthy subjects, fasting total plasma DA was below 0.1 μM and most non-fasting samples contained submicromolar concentrations (46). By contrast, children receiving a high-MCT ketogenic diet had a reported mean plasma DA concentration of 27.0 mg/L, approximately 157 μM (47), showing that total systemic concentrations can overlap the experimental range under a specialized dietary exposure. That comparison does not establish free drug levels in synovial fluid or support a dosing regimen. For local delivery, a purely illustrative 10–20 mL distribution volume at 100–200 μM would contain approximately 0.17–0.69 mg DA, but actual joint exposure will depend on formulation, ionization, albumin binding, membrane partitioning, uptake, metabolism, dilution, clearance, and residence time. No intra-articular pharmacokinetic or tolerability data were generated here; the tested concentrations should therefore be regarded as proof-of-concept exposures rather than clinically established targets.

DA-associated increases in 17(S)-HDHA and MaR2 are of interest because these mediators are linked to resolution pathways. However, our data do not show that intracellular PGE2 retention directly caused their formation. The relationship could reflect parallel effects of DA on fatty-acid metabolism, lipoxygenase activity, PPAR signaling, cellular stress, or other pathways. Establishing causality will require time-resolved isotope tracing, measurement of intracellular prostaglandin metabolites, direct manipulation of MRP4, and demonstration that the oxylipin changes disappear when prostaglandin retention is prevented.

The biological desirability of redistributing PGE2 is also context dependent. Extracellular PGE2 can amplify synovial inflammation and nociception, yet PGE2 has been reported to provide autocrine antihypertrophic feedback during mesenchymal stromal cell chondrogenesis, and cyclooxygenase-2 inhibition can compromise that response (18). More recently, inhibition of the PGE2-degrading enzyme 15-PGDH increased PGE2 signaling and promoted cartilage regeneration in preclinical OA models (19). These observations caution against equating lower extracellular PGE2 with universal joint benefit. DA may preserve total intracellular prostaglandin pools relative to global cyclooxygenase blockade, but intracellular retention does not necessarily preserve extracellular EP-receptor signaling and cannot be assumed to be chondroprotective. The net effect could differ between synovium, cartilage, immune cells, and sensory neurons. The present synoviocyte monoculture did not assess chondrocyte hypertrophy, cartilage matrix synthesis, EP-receptor signaling, or tissue repair.

The clinical relevance of the combined DA-Dex effect is rooted in its potential as a steroid-sparing therapeutic strategy. Glucocorticoids inhibit the production of inflammatory prostaglandins by regulating PLA2 and cyclooxygenase/PGE isomerase (43). Although this upstream activity accounts for the efficacy of Dex, frequent or high-dose intra-articular administration can negatively affect cellular and matrix homeostasis (29, 32, 33). DA appears to complement Dex by acting downstream of synthesis, specifically at the level of prostaglandin export. Functionally, the combination integrates upstream synthesis inhibition with downstream transport restriction. This dual-target strategy allows for significant suppression of extracellular prostaglandins while minimizing the Dex dosage necessary to achieve the desired therapeutic effect. Even so, steroid-sparing efficacy has not been demonstrated *in vivo*, and the present fixed-background experiment cannot define an optimal dose ratio or therapeutic window. Future studies should compare the combination with clinically used corticosteroid exposures in synovial explants and joint models while monitoring cartilage outcomes.

Although Dex was selected here to test a corticosteroid-sparing strategy, DA could also potentially be combined with a disease-modifying osteoarthritis drug or another cartilage-directed intervention. Such an approach could couple modulation of extracellular prostaglandin signaling with a therapy directed toward cartilage catabolism, matrix restoration, chondrocyte phenotype, or subchondral bone. However, DMOAD candidates act through heterogeneous pathways, and the context-dependent functions of PGE2 mean that additivity or synergy cannot be assumed. Candidate DA-DMOAD combinations should therefore be tested directly in multi-donor chondrocytes, synoviocytes, and joint-tissue explants using inflammatory, catabolic, matrix, viability, and formal drug-interaction endpoints.

Several limitations are central to interpretation. First, all synoviocyte experiments used one OA donor. Independent wells or cell pellets measure within-donor experimental variation but cannot capture inter-patient heterogeneity in OA phenotype, sex, age, medication exposure, disease stage, or transporter expression. The findings therefore cannot be generalized to the broader OA population. Second, the transport assay used DHEAS rather than PGE2, and docking does not prove binding. Third, intra-articular pharmacokinetics, formulation behavior, and safety were not assessed. Finally, a synoviocyte monoculture does not reproduce biomechanical loading, cartilage-synovium crosstalk, immune-cell interactions, or joint clearance.

Future work should prioritize prospectively designed multi-donor validation with donor treated as the biological unit; direct PGE2 transport assays in MRP4 vesicles; and a replicate-level DA-by-Dex interaction matrix. Time-resolved lipid flux studies should test the class-switching hypothesis. Chondrocytes, mixed synovial explants, and osteochondral models are needed to determine whether reduced synovial prostaglandin signaling can be achieved without impairing cartilage homeostasis. Finally, formulation, intra-articular pharmacokinetic/pharmacodynamic, and local safety studies are required before translational dosing can be proposed.

## 5 Conclusion

In primary OA synoviocytes from one donor, DA reduced extracellular prostaglandin accumulation, increased intracellular PGE2, inhibited MRP4-mediated DHEAS probe transport, and was associated with increased 17(S)-HDHA and MaR2. These convergent findings are consistent with an MRP4-associated transport hypothesis, but they do not directly demonstrate inhibition of PGE2 transport or establish that prostaglandin retention caused resolution signaling. A fixed, minimally active Dex background enhanced the observed suppression of extracellular PGE2, but formal potentiation or synergy was not demonstrated. The study should therefore be regarded as preliminary proof of concept. Multi-donor validation, direct PGE2 transport and genetic MRP4 experiments, chondrocyte and tissue-level studies, and intra-articular pharmacokinetic and safety evaluation are required before therapeutic claims can be made.

## Data Availability

The datasets presented in this study can be found in online repositories. The names of the repository/repositories and accession number(s) can be found below: Data [Williams, Jason (2026), “OA Synoviocyte Lipidomic Dataset”, Mendeley Data, V1, doi: 10.17632/vw5jd4n5dr.1].

## Supplementary material

SUPPLEMENTARY FIGURE S1 Specialized pro-resolving mediator pathway overview. Specialized Pro-resolving Mediators (SPMs) are synthesized from Omega Polyunsaturated fatty acids (PUFAs). AA, arachidonic Acid; DHA, docosahexaenoic acid; EPA, eicosapentaenoic acid; LOX, lipoxygenase; COX, cyclooxygenase; CYP450, cytochrome p450; HDHA, hydroxy-docosahexaenoic acid.

SUPPLEMENTARY FIGURE S2 High-confidence oxylipins identified by UHPLC-MS 34 high-confidence oxylipins were measured. Oxylipins were quantified based on their relative abundance using Peak Intensity, as calculated by the Area Under the Curve. Oxylipins were detected in all six test groups. All groups except “Media Only” received IL-1*β* and treatment.
