# Supplementary material for: Decanoic acid reduces extracellular prostaglandin accumulation, inhibits MRP4 transport, and enhances low-dose dexamethasone effects in IL-1β-stimulated human osteoarthritis synoviocytes
Source: Front Med (Lausanne). 2026 Oct 20;13:1876378. doi: 10.3389/fmed.2026.1876378 (PMC13727426; doi:10.3389/fmed.2026.1876378)
Supplement: Supplementary file 1 [file Datasheet1.docx]

Supplementary Material

# 1 Supplementary Methods

# Inside-out Membrane Vesicle Test System

Vesicular transport assays were performed with inside-out membrane vesicles prepared from cells overexpressing human ABC (efflux) transporters. The transporters were expressed by SOLVO Biotechnology in mammalian (HEK293) cells. The mammalian cells were stably transfected with the ABC transporter of interest. The assays were conducted according to the actual SOLVO VT assay protocols (PR-ASY-VT-General Protocol for Vesicular Transport Inhibition Assays).

Parameters of the vesicular transport assays are listed in Table 1.

Table S1.	Vesicular transport assay parameters

| Transporter | Applying protocol | Protein content/well | Incubation time | Probe substrate | Reference inhibitor | Buffer |
| --- | --- | --- | --- | --- | --- | --- |
| human MRP4 (HEK293) | DS-ASY-VT-MRP4-HEK293-DHEAS | 50 µg | 1.5 min | DHEAS  (0.5 µM) | MK-571  (150 µM) | Transport buffer with sucrose |

#### 1.1.1.1 Experimental method for vesicular transport inhibition assay

The membrane vesicle suspension was mixed with the ice-cold transport buffer, then the probe substrate solution was added. This reaction mixture was distributed at 50 µL per well (total protein: 50 µg/well) and pre-incubated with Decanoic acid or the positive control at 1.5-times the final assay concentrations for 15 minutes at 37 °C. The reactions were initiated by the addition of 25 µL per well pre-warmed 12 mM MgATP (or 12 mM AMP as a background control) to the 50 µL per well reaction mixtures, diluting the ATP and AMP to 4 mM and Decanoic acid to the final assay concentrations. Incubations were conducted under assay parameters specified in established protocols listed in Table S1. Reactions were quenched by the addition of 200 µL of ice‑cold washing buffer and immediate filtration via glass fiber filters mounted to a 96-well plate (filter plate). The filters were washed (5 × 200 µL of ice‑cold washing buffer), dried and the amount of substrate inside the filtered vesicles was determined by liquid scintillation counting.

Treatment groups applied in the vesicular transport inhibition assays are listed in Table S2.

Table S2.	Vesicular transport inhibition assay conditions, controls

| Treatment groups in 96-well plate format | |
| --- | --- |
| Transporters: MRP4 | |
| Assay Conditions | Controls |
| - • Decanoic acid: Solvent control, at 7.81, 15.63, 31.25, 62.5, 125.0, 250.0 and 500.0 µM - 4 mM Mg-ATP or 4 mM AMP - No. of replicates: 3 | - 4 mM Mg-ATP or 4 mM AMP - Functionality control:   - Solvent control (100% activity values)*   - Reference inhibitor (positive control for inhibition) - No. of replicates: 3 |

*applied if TA and inhibitor solvents or dilution factors are different.

#### 1.1.1.2 Controls for vesicular transport inhibition assay

- Incubation with AMP provided background activity values for all data points.
- Incubations with a respective probe substrate (100% activity values) in the presence and absence of a reference inhibitor were performed for each efflux transporter to confirm the transporter function.

*Calculations and data processing*

VT Inhibition Assays

In the vesicular transport inhibition experiments, values are presented on a relative scale, with 100% defined as transport in the presence of the solvent and without TA (no inhibition), and 0% defined as probe substrate accumulation in the absence of transporter activity. Results will be plotted as inhibition of ATP-dependent/transporter-specific transport [relative activity (%)] versus TA concentration (μM). Maximal inhibition is defined as the maximum effect observed in the concentration range investigated in the experiment. IC50 is defined as the concentration required to inhibit the transport of the probe substrate by 50%. If transport stimulation is observed, the effect will be reported as net stimulation greater than 100%.

IC50 value calculations will be performed using a 4-parameter equation: Curve fitting (non-linear regression; log (inhibitor) vs. response – variable slope).

$$Y = Bottom+\frac{Top-Bottom}{(1+{10}^{\left( LogIC50-X \right)*Hillslope)})}$$

Legend:	**X**: log of concentration

**Y**: response

**Top** and **Bottom**: plateaus in same units as Y

**log IC_50_**: same log units as X

**HillSlope**: Slope factor Hill slope, unitless

# 2 Supplementary Methods

**Lipidomic Analysis**

**Oxylipin Standards used in study**

12(13)-DiHOME d4

12(S)-HETE-d8

13(S)-HODE-d4

13-14-dihydro-15-keto Prostaglandin D2-d9

13-OxoODE-d3

15(S)-HEPE-d5

15(S)-HETE-d8

17(S)-HDHA-d5

5(S)-HETE-d8

6-keto Prostaglandin F1a-d4

9(10)-DiHOME d4

9(S)-HODE-d4

9-OxoODE-d3

a-Linolenic Acid-d5

Arachidic Acid-d2

Arachidonic Acid-d8

Decanoic Acid-d2

Dihomo-y-Linolenic Acid-d6

Docosahexaenoic Acid-d5

Docosapentaenoic Acid-d5

Eicosapentaenoic Acid-d5

Heptadecanoic Acid-d3

Lauric Acid-d2

Leukotriene B4-d4

Linoleic Acid-d11

Myristic Acid-13C

Oleic Acid-13C

Palmitelaidic Acid-d13

Palmitic Acid-d5

Pentadecanoic Acid-d3

Prostaglandin A2-d4

Prostaglandin D2-d9

Prostaglandin E2-d9

Stearic Acid-d4
